# Enhancing Drug Delivery and Efficacy in Stroke via Supramolecular Nanoparticle Systems

**DOI:** 10.1021/acsomega.6c04753

**Published:** 2026-10-13

**Authors:** Xing Yu Huang, Ming Yang Xu, Meng Zhang, Xin Lu, Jian Bin Tang, Bing Chun Yan, Jia Cheng Wang

**Affiliations:** † School of Traditional Chinese Medicine, Faculty of Medicine, 38043 Yangzhou University, Yangzhou 225009, PR China; ‡ Key Laboratory of the Jiangsu Higher Education Institutions for Integrated Traditional Chinese and Western Medicine in Senile Diseases Control (Yangzhou University), Yangzhou 225009, PR China; § Department of Neurology, Yangzhou University Affiliated Taixing Hospital, Taizhou 225400, PR China; ∥ Changzhou Center for Disease Control and Prevention, Changzhou 213022, PR China

## Abstract

The pathogenesis of ischemic stroke involves multiple pathological links, such as blood–brain barrier (BBB) disruption, oxidative stress, and neuroinflammation, making comprehensive intervention via a single drug exceedingly difficult. In this study, we constructed a supramolecular coassembly nanoparticles (UA-Gen NPs) composed of Ursolic acid (UA) and Genistein (Gen), which self-assemble into a stable supramolecular structure driven by intermolecular hydrogen bonding and π-π stacking. This nanoplatform not only significantly improved drug hydrophilicity but also achieved a transformation from simple physical mixing to structural integration. In vitro profiles revealed a distinct pH-responsive and sequential release pattern of the system (“fast-Gen/slow-UA”). Pharmacokinetic studies further demonstrated that this coassembly significantly prolonged blood circulation and synchronized the fates of the two drugs in vivo. In a mouse middle cerebral artery occlusion (MCAO) model, UA-Gen NPs exhibited superior efficacy in alleviating BBB leakage, reducing infarct volume, and improving neurobehavioral outcomes compared to their physical mixture. Serological analyses confirmed its regulation of oxidative stress and systemic inflammation, yielding optimal neuroprotection. At the protein expression level, the nanotherapy significantly inhibited Keap1, upregulated Nrf2, and restored Occludin expression. Altogether, by utilizing drug molecules themselves as structural building blocks, this study engineered a supramolecular nanotherapeutic agent featuring microenvironment-responsive release behavior and sequential drug delivery. This work provides a feasible research strategy to overcome the challenges of combination therapy in complex diseases.

## Graphical abstract

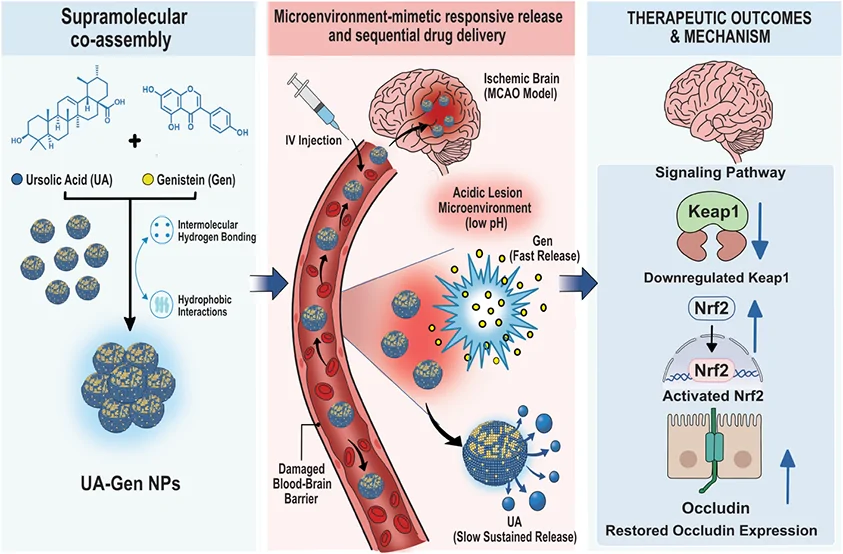

## 1 Introduction

Ischemic stroke poses a tremendous global public health challenge due to its high mortality and disability rates.^1,2^ Currently, the time window for clinical treatment is extremely limited, and there is a lack of neuroprotective drugs that can effectively protect the blood–brain barrier (BBB) and simultaneously target multiple injury mechanisms.^3,4^ Therefore, developing novel therapeutic strategies that enable efficient drug delivery and improve the regulation of complex pathological networks poststroke is of urgent clinical significance.

Natural bioactive components have shown broad prospects in the treatment of stroke because of their multitargeted effects and good safety.^5,6^ Among these, the combination of Ursolic acid (UA) and Genistein (Gen) shows considerable potential because UA exhibits powerful anti-inflammatory and antioxidant properties, while Gen is a well-known tyrosine kinase inhibitor that can regulate apoptosis and inflammatory signaling pathways.^7–10^ Theoretical predictions indicate that the two compounds can jointly intervene in key pathological processes after stroke, including oxidative stress, cell apoptosis, and blood–brain barrier disruption, with complementary regulatory effects. However, both UA and Gen are hindered by inherent drawbacks such as poor aqueous solubility, low bioavailability, and unfavorable in vivo distribution. More importantly, their distinct physicochemical properties make it exceptionally difficult to achieve synchronized delivery and codistribution in vivo, profoundly limiting the realization of their theoretical combined intervention potential.^11,12^

Nanodelivery technology has provided the possibility to overcome the above bottlenecks.^13–15^ However, traditional loaded nanocarriers (e.g., liposomes, polymeric micelles) generally suffer from limited drug loading efficiency and are unable to precisely control the release kinetics of drugs with distinct properties.^16–18^ In recent years, the emerging supramolecular drug coassembly nanotechnology has provided a new strategy for achieving codelivery.^19,20^ Including multiple treatment combinations: ursolic acid paired with 18β-glycyrrhetinic acid for malaria, astaxanthin (AST) and geniposide (Gen) for atherosclerosis, and glycyrrhizin (GA) with puerarin (PUE) for corneal alkali burns. These bioactive molecules directly form coassembled nanoparticles through noncovalent interactions, such as hydrophobic interactions, van der Waals forces and hydrogen bonds, which avoids the use of exogenous carrier materials.^21–23^ However, there have been no reports on exploiting specific intermolecular interactions between UA and Gen to fabricate pH-responsive supramolecular coassembled nanoparticles for ischemic stroke therapy. Therefore, developing such nanosystems to achieve efficient collaborative delivery is an attractive challenge.

Motivated by this, the present study proposes a rationally designed integrated combination therapeutic approach. The superiority of this system was systematically demonstrated through a complete chain of evidence: 1) The coassembly mechanism is elucidated from the molecular interaction level to the morphological structural level. 2) Its pH-responsive drug release property and favorable in vivo delivery profile were demonstrated through in vitro release and pharmacokinetic investigations. 3) Its neuroprotective, anti-inflammatory, antioxidant, and blood–brain barrier repairing effects far exceed simple physical mixtures and have been thoroughly validated in MCAO mouse models. 4) Combined with network pharmacology predictions and experimental verification, the regulatory effects of UA-Gen NPs on key pathway-related proteins, such as Keap1, Nrf2, and Occludin, were investigated. This work not only provides a highly efficient UA-Gen nanoformulation for stroke therapy but also offers a referential strategy to develop cooperative coassembly delivery systems based on natural products.

## 2 Materials and Methods

### 2.1 Materials

Ursolic acid (UA, ≥98%) and Genistein (Gen, ≥98%) were purchased from Nanjing Chunqiu Bioengineering Co., Ltd. Boric acid, iodine, and potassium iodide were obtained from Shanghai Aladdin Biochemical Technology Co., Ltd. DMEM high-glucose medium and fetal bovine serum (FBS) were obtained from Gibco. ELISA assay kits for IL-6, IL-1β, TNF-α, SOD, GSH-Px, and MDA detection were all purchased from Nanjing Jiancheng Bioengineering Institute.

Antibodies: Phospho-NRF2-S40 Rabbit mAb (Nrf2, AP1133, Abclonal Technology Co., Ltd., China), keap1 (keap1, ab227828, Abcam plc, UK), occludin and Anti-VEGFA antibody (VEGFA, ab46154, Abcam plc, UK), β-Actin (13E5) Rabbit mAb (β-Actin, 4970S, Cell Signaling Technology Inc., USA). GFAP antibody (bs-0199R) purchased from Bioss and the corresponding secondary antibodies were purchased from Proteintech. All other chemical reagents used in the experiments were of analytical grade.

Healthy adult male SPF ICR mice (6–8 weeks old, weighing 25–30 g) were purchased from Yangzhou University. All the mice were kept in an SPF animal house with free access to food and water under a 12 h light/dark cycle. All animal experiments comply with the regulations of the Animal Ethics Committee of Yangzhou University.

### 2.2 Preparation and Characterization of UA-Gen Coassembled Nanoparticles

#### 2.2.1 Preparation of Nanoparticles

UA-Gen nanoparticles (UA-Gen NPs) were prepared by ultrasonic emulsification-self-assembly. The brief procedure was to accurately weigh proper amount of UA and Gen and dissolve them in proper amount of dichloromethane to form an organic phase. Under the condition of ice bath, the organic phase was slowly injected into a certain volume of 2.5% PVA solution for ultrasonic emulsification. Then, the emulsion was transferred to PVA solution containing 0.3% and stir overnight until the organic solvent was completely removed. The obtained nanosuspension was fully washed with ultrapure water (removing residual PVA), resuspended in ultrapure water or PBS, and stored at 4 °C for later use. Different NPs were prepared by similar methods. The physical mixing (Mix) solution was prepared by directly dissolving the same amount of UA and Gen in a solution containing 10% cosolvent (DMSO + propylene glycol + PEG 400). The residual PVA content in nanoparticles was determined using iodine-boric acid spectrophotometry method.

#### 2.2.2 Physicochemical Characterization of Nanoparticles

##### 2.2.2.1 Particle Size, Polydispersity Index, and Zeta Potential

The hydrated hydrodynamic diameter, polydispersity index (PDI) and surface Zeta potential of the nanoparticles were measured at 25 °C by a Malvern particle size analyzer (DLS, Zetasizer ZS-90, Malvern, UK). Each sample was measured in triplicate.

##### 2.2.2.2 Morphological Observation

The freeze-dried powder of the samples was placed on conductive adhesive and subjected to gold sputtering treatment. The microstructure was observed using a scanning electron microscope (SEM, GeminiSEM 300). A 2 μL aliquot of the sample dispersion was dropped onto a carbon-coated copper grid, and the microstructure was observed using a transmission electron microscope (TEM, Tecnai 12).

##### 2.2.2.3 Surface Hydrophilicity and Hydrophobicity

The water contact angles of the compressed tablets of UA, Gen and UA-Gen NPs were measured using a contact angle goniometer (Biolin, Theta Flex).

##### 2.2.2.4 Stability Analysis

The UA-Gen NPs suspension was stored at 4 °C. Samples were collected at different storage time points (0, 1, 7, 15, 30, and 60 days), and the changes of particle size and PDI were measured by DLS to evaluate the physical stability. If the deviation in particle size from the initial value was ≤10% and the PDI remained ≤0.3 throughout the storage period, the freeze-dried formulation was deemed to have passed the short-term stability evaluation based on particle size and PDI under the aforementioned accelerated conditions.

#### 2.2.3 Analysis of Molecular Interactions

##### 2.2.3.1 Fourier Transform Infrared Spectroscopy (FTIR)

The chemical structures of the samples were analyzed in the wavenumber range of 4000–400 cm^–1^ using a Fourier transform infrared spectrometer (Shimadzu, FTIR-21).

##### 2.2.3.2 X-ray Diffraction (XRD) Pattern Analysis

X-ray diffractometer (Bruker, D8 Advance) was used to analyze the crystal structure in a set scanning range (5–80 2 θ).

##### 2.2.3.3 Thermal Stability Analysis

The thermal stability of the samples was analyzed using a thermogravimetric analyzer (PerkinElmer, Pyris 1 TGA) over a temperature range of 30–800 °C with a heating rate of 10 °C/min.

#### 2.2.4 Study on pH-Responsive Release Characteristics

##### 2.2.4.1 In Vitro Release Kinetics

The pH-responsive release characteristics of UA-Gen NPs were studied by dialysis bag method. The UA-Gen NPs suspension was placed in a dialysis bag (molecular weight cutoff, MWCO 3.0 kDa) and immersed in 500 mL of phosphate-buffered saline (PBS) release medium with different pH values (7.4, 6.5, 5.0). The medium contained 0.5% (w/v) Tween 80 to prevent drug supersaturation. The release experiment was carried out under constant temperature shaking conditions at 37 °C and 100 rpm. At predetermined time points (10 min, 20 min, 30 min, 1 h, 2 h, 4 h, 6 h, 8 h, 12 h, 24 h), 200 μL of the dialysate was collected, and an equal volume of fresh medium was replenished immediately. The concentrations of UA and Gen were determined separately by HPLC (chromatographic column: C18. Mobile phase: acetonitrile: 0.1% formic acid water = 70:30), and their respective cumulative release percentages were calculated. The release data were fitted with different kinetic models (zero order, first order, Higuchi, Korsmeyer-Peppas).

##### 2.2.4.2 Zeta Potential-pH Profile

UA-Gen NPs were dispersed in PBS solutions with different pH values (5.0, 5.5, 6.0, 6.5, 7.0, 7.4), and their surface charges were measured using a zeta potential analyzer. The curve of Zeta potential changing with pH was drawn to determine the isoelectric point (IEP), thereby providing a mechanistic explanation for the release behavior.

### 2.3 Evaluation of Biocompatibility and Safety

#### 2.3.1 Hemolysis Test

Fresh anticoagulated blood was collected from healthy rabbits, and red blood cells (RBCs) were obtained by centrifugation. After washing, the RBCs were prepared into a 2% RBC suspension. Different concentrations of UA-Gen NPs were incubated with the RBC suspension, followed by centrifugation. The absorbance of the supernatant was measured at 540 nm. Deionized water (100% hemolysis) and normal saline (0% hemolysis) were used as controls to calculate the hemolysis rate.

#### 2.3.2 Evaluation of Cellular Safety Assessment of Nanoparticles

The cytotoxicity assay was performed using the RAW 264.7 cell line, bEnd.3 cell line, and HT22 cell line, respectively. The cells were seeded into 96-well plates, and after adherence, different concentrations of UA, Gen, and UA-Gen NPs were added separately. Following 24 h of incubation, the cell viability was determined by the MTT assay. Healthy mice (n = 5 per group) were treated with triple therapeutic dose of UA-Gen NPs. Serum was separated from collected blood samples, and the levels of AST, ALT, urea and sCr were quantified by biochemical kits to detect hepatorenal function.

### 2.4 In Vivo Pharmacodynamic Study

#### 2.4.1 Pharmacokinetic Study

##### 2.4.1.1 Animal Grouping and Administration

Sprague–Dawley (SD) rats were randomly divided into two groups (n = 3 per group), and the equivalent dosage of UA-Gen NPs suspension or UA + Gen physical mixed solution was injected into the tail vein, respectively. Blood samples were collected from the retro-orbital venous plexus before administration and at a series of precise time points postadministration (0, 0.2, 0.5, 1, 2, 3, 4, 6, 8, 12, 24, 48 h). After the plasma samples were processed, the plasma concentrations of UA and Gen were determined by HPLC. The main pharmacokinetic parameters were calculated using DAS software.

#### 2.4.2 MCAO Model Establishment and Grouping

The intraluminal filament method was used to establish the middle cerebral artery occlusion (MCAO) model for simulating ischemia-reperfusion injury.

We established a mouse cerebral ischemia-reperfusion MCAO model via the modified intraluminal filament method. Commercial sterile ready-to-use filaments were used: shaft diameter 0.16 ± 0.01 mm, hemispherical tip diameter 0.22 ± 0.01 mm, total length 33–38 mm, with the front 10 mm covered by poly-l-lysine. The filament was placed in the internal carotid artery to occlude the middle cerebral artery for 20 min before reperfusion. Sham mice underwent vessel exposure only without filament insertion.

Mouse core temperature was maintained at 37.0 ± 0.5 °C with heating pads and rectal probes during surgery and the subsequent 3 days. Cortical cerebral blood flow was detected by laser Doppler flowmetry. Mice with postinsertion blood flow <20% baseline and reperfusion recovery >50% baseline were defined as successful models. Animals with intraoperative hemorrhage, vessel rupture, anesthetic death, postoperative severe seizures, early death or unqualified blood flow were eliminated. The 3-day mortality of MCAO mice was 12%–18%, while no sham mice died from surgery. Healthy male mice aged 8–10 weeks weighing 25–30 g with 7 days of adaptive feeding were enrolled.

Mice were randomly divided into four groups: sham operation group (Sham), model group (MCAO), UA + Gen physical mixture treatment group (UA + Gen Mix), and UA-Gen nanoparticles treatment group (UA-Gen NPs). Corresponding drugs or an equal volume of normal saline were administered through tail vein at the initiation of reperfusion and once daily thereafter for a consecutive 3 days.

All procedures including drug administration, neurological scoring and brain tissue detection were performed under single-blind conditions throughout the experiment.

#### 2.4.3 Neurological Function Scoring (mNSS)

24 h after operation, the mice were tested by researchers who were unaware of the grouping, such as neurological behavioral score (BBT), improved neurological deficit score (mNSS) and rotarod test.

#### 2.4.4 Assessment of Cerebral Infarct Volume and Blood–Brain Barrier Permeability

##### 2.4.4.1 Assessment of Cerebral Infarct Volume by TTC Staining

After the final behavioral test, the mice were euthanized and their brains were harvested. The collected fresh mouse brains were cut into coronal sections with uniform thickness along the sagittal axis, and stained in 2% TTC staining solution. The slices were flipped every approximately 10 min to ensure uniform staining. The percentage of infarct area was calculated using ImageJ software.

##### 2.4.4.2 Assessment of Blood–Brain Barrier Disruption via Evans Blue (EB) Extravasation Assay

The middle cerebral artery occlusion (MCAO) model was established using the standard intraluminal filament method, and different interventions were administered according to Section 2.4.2. Four hours prior to euthanasia, 2% Evans Blue solution was slowly injected via the tail vein (successful injection was confirmed by the blue discoloration of the mice’s ear marginal veins). Before execution, the mice were anesthetized and perfused to remove the intravascular dye. Finally, the mouse brains were harvested, and photographs of the whole brains and stained thick sections were taken.

#### 2.4.5 Histopathological and Immunohistochemical Analysis

Brain tissues were collected from experimental animals in each group, fixed in 4% paraformaldehyde for 24 h, and then subjected to gradient dehydration, transparency treatment, wax infiltration and embedding sequentially. Finally, 5 μm paraffin sections were prepared and dried. Hematoxylin-eosin (H&E) staining was performed separately to observe the general pathological morphology under an optical microscope. Immunohistochemical staining for GFAP was conducted, and the morphology, number, and GFAP expression intensity of astrocytes in the sections of each group were observed under the microscope.

#### 2.4.6 Determination of Serum Biochemical Indicators

Serum samples were collected from animals in each group. Strictly following the instructions provided with the corresponding ELISA kits, the levels of inflammatory factors (IL-6, IL-1β, TNF-α) and the oxidative stress indicators (SOD activity, GSH and MDA contents) were determined.

#### 2.4.7 Western Blot Analysis

Total protein was extracted from Cortical tissues of the ischemic hemisphere and quantified by BCA method. The same amount of protein was loaded and transferred to PVDF membrane after SDS-PAGE electrophoresis. After blocking with 5% nonfat milk, the membranes were incubated separately with primary antibodies against Keap1, Nrf2, Occludin, and the internal reference (β-actin) (dilution ratio, e.g., 1:1000) at 4 °C overnight. The membranes were washed three times with TBST, followed by incubation with HRP-conjugated secondary antibody (Goat Anti-Rabbit IgG H&L) for 1 h at room temperature. Protein bands were visualized via ECL chemiluminescence, and the gray values of the bands were analyzed using ImageJ software.

### 2.5 Network Pharmacology and Transcriptomics

The CAS numbers of UA and genistein were converted by PubChem. The corresponding targets of UA and genistein were obtained from the DrugBank, TCMSP, and STITCH databases. Meanwhile, stroke-related gene/protein targets were collected from the OMIM, GeneCards, and TTD disease databases.

The “ursolic acid/genistein-target-stroke” interaction network was constructed using Cytoscape software. Core targets regulating this network were screened via topological analysis.

GO functional annotation (gene function) and KEGG pathway enrichment analysis (signaling pathway) were performed on the core targets to clarify the biological processes in which they are involved.

### 2.6 Statistical Analysis

All data were expressed as mean ± standard deviation (Mean ± SD) and statistically analyzed with GraphPad Prism 10.

One-way analysis of variance (ANOVA) was applied for comparisons among multiple groups, followed by Tukey’s posthoc test. Student’s *t* test was applied for comparisons between two groups. A value of *P* < 0.05 was considered statistically sinificant (*p < 0.05, **p < 0.01, ***p < 0.001).

## 3 Results and Discussion

Ischemic stroke exhibits complex pathology, where blood–brain barrier disruption, oxidative stress, and inflammatory responses jointly mediate neuronal damage. UA has antioxidant and anti-inflammatory effects, while Gen can inhibit apoptosis and regulate angiogenesis. However, a single drug cannot adequately counteract the complex pathological network of ischemic stroke, while combination therapies face challenges including poor aqueous solubility, divergent in vivo pharmacokinetic behaviors, and inefficient drug accumulation in cerebral tissue. This study proposed and validated a novel strategy: coassembling UA and Gen with complementary molecular regulatory effects to construct pH-responsive nanoparticles (UA-Gen NPs) that can respond to the pathological microenvironment and realize controlled drug release.

### 3.1 Morphological and Structural Evolution Induced by Coassembly

First, we verified the transformation of UA and Gen from individual entities to a symbiotic integrated whole.

Under scanning electronic microscopy (SEM), the original micron-sized Gen with dense parallel needlelike morphology and UA without specific regular structure disappeared. Instead, small-sized irregular nanoparticles were observed (Figure 1A), indicating a significant change in the aggregation state of the two compounds after combination. The transmission electronic microscope (TEM) characterization of UA-Gen NPs confirmed that they formed a regular spherical composite structure, which were completely different from their original morphologies in the individual state (solid bundle-like and irregular core–shell structures) (Figure 1B). This was not a simple physical mixture, but a profound “re-creation” driven by intermolecular forces. Dynamic light scattering (DLS) analysis demonstrated that the hydrodynamic diameter of UA-Gen NPs (approximately 251 nm) was significantly reduced compared with that of UA NPs (approximately 362 nm). Moreover, the surface charge of UA-Gen NPs (−12.3 ± 0.96) exhibited a significant change compared with that of the individual components, indicating the change of their surface properties (Figure 1C, D). The results of the long-term stability test showed that the particle size and polydispersity index (PDI) of UA-Gen NPs remained stable after storage at room temperature for 60 days, showing excellent storage stability (Figure 1E). Crucially, water contact angle measurements showed that the hydrophilicity of UA-Gen NPs (43.5°) was much better than that of the hydrophobic UA (110.8°) and Gen (85.3°) (Figure 1F), and this property laid a solid foundation for their excellent biocompatibility and dispersibility.

**Figure 1:**
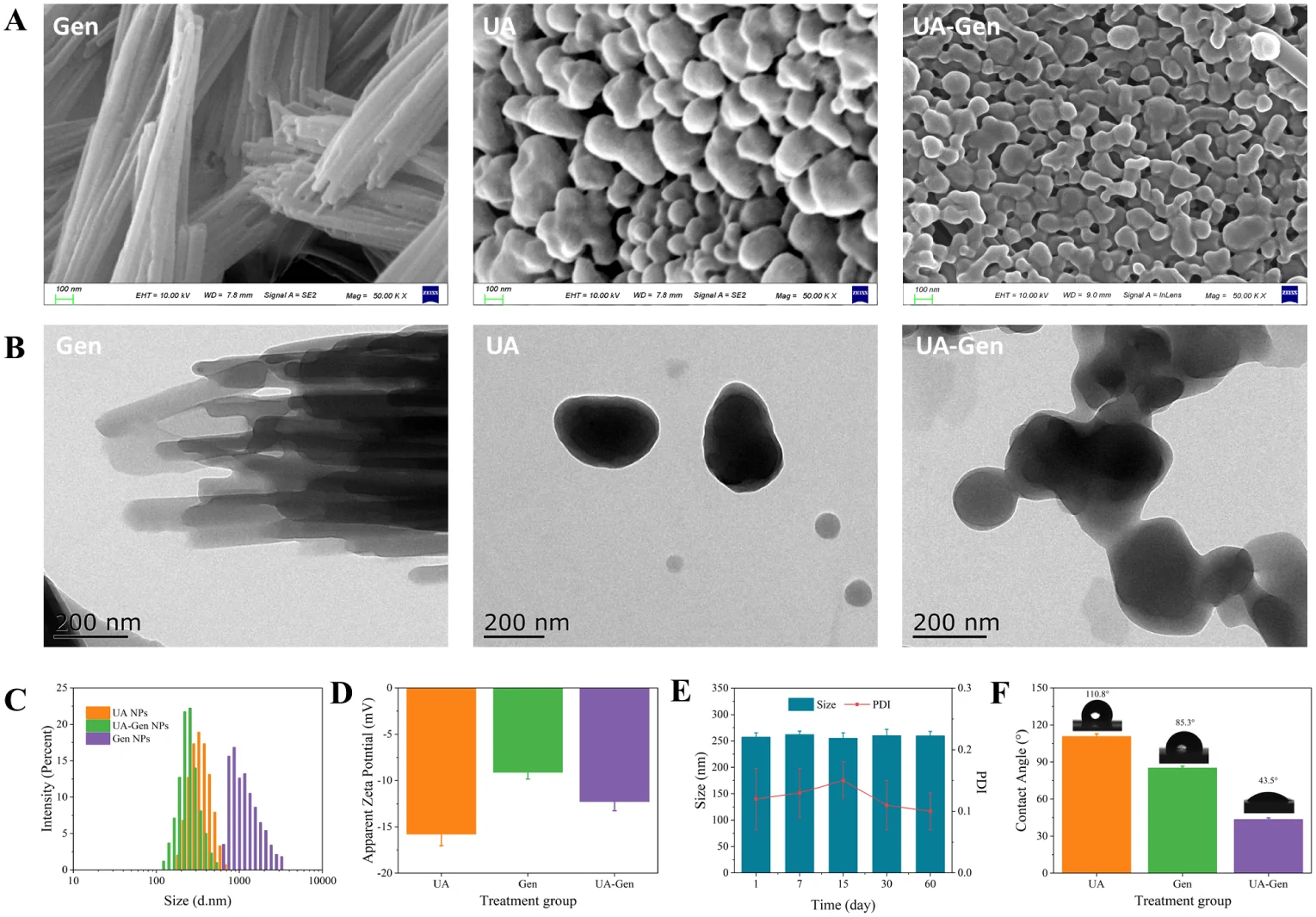
Structural and interfacial physicochemical characteristics of Gen NPs, UA NPs, and UA-Gen NPs. A. SEM images: Gen presented as fibrous morphology (micron-scale), UA existed as irregular spheres (500 nm), while UA-Gen NPs showed uniform nanoparticles (nanoscale). B. TEM images: Gen exhibited solid bundle-like fibers, UA displayed irregular core–shell nanoparticles, and UA-Gen NPs appeared as spherical nanoparticles (200 nm). C. DLS particle size distribution profiles: Hydrodynamic diameter distribution of aqueous dispersions of UA NPs, Gen NPs, and UA-Gen NPs with different mass ratios. D. Zeta potential analysis: Surface charge values of UA NPs, Gen NPs, and UA-Gen NPs. E. Storage stability of UA-Gen NPs (stored at room temperature in the dark): Changes in particle size (blue columns) and polydispersity index (PDI, red line) within 60 days, demonstrating favorable storage stability. F. Water contact angle measurements: Contact angle values and droplet morphologies (insets) of UA (110.8°), Gen (85.3°), and UA-Gen NPs (43.5°).

Together, these results prove that we have successfully achieved the molecular coassembly of UA and Gen. This process was not a simple physical mixture, but a bottom-up “de novo construction” driven by noncovalent interactions. Such structural integration fundamentally resolved the solubility and delivery challenges of UA and Gen caused by their hydrophobicity, thereby creating a prerequisite for the subsequent achievement of synchronous pharmacokinetics and combinatorial pharmacodynamic effects.

### 3.2 Molecular Interaction Mechanisms

To elucidate the driving forces of coassembly, we conducted an in-depth investigation into its molecular mechanisms and functional responses. Characteristic peaks in Fourier transform infrared spectroscopy (FTIR) spectra (Figure 2A) showed that the spectrum of the UA + Gen physical mixture (UA + Gen Mix) clearly exhibited the coexistence of the aliphatic hydroxyl stretching vibration of UA (∼3525 cm^–1^) and the phenolic hydroxyl stretching vibration of Gen (∼3412 cm^–1^) in the 3500–3400 cm^–1^ region, which appeared as the superposition of two independent peak positions. In the characteristic spectrum of UA-Gen NPs, the characteristic O–H peak of UA at 3525 cm^–1^ completely disappeared. Meanwhile, the stretching vibration peak of its carboxylic carbonyl group (C=O) showed a significant bathochromic shift from 1717 cm^–1^ to 1691 cm^–1^. The carboxyl group of UA acts as an excellent hydrogen bond acceptor, and the electron cloud density of its C=O bond decreases due to its involvement in the formation of stronger hydrogen bonds, leading to a reduction in vibration frequency. In contrast, the hydroxyl group of UA serves as a hydrogen bond donor and is fully incorporated into the newly formed hydrogen bond network. Meanwhile, the phenolic hydroxyl O–H peak of Gen (∼3413 cm^–1^) was preserved but with a slight peak shape change, suggesting that some of its phenolic hydroxyl groups might act as hydrogen bond donors to bind with the carboxyl oxygen or ether oxygen atoms of UA. Furthermore, in the spectrum of the UA + Gen physical mixture (UA + Gen Mix), both the aromatic ring skeleton vibration of Gen (νC = C_ar_, ∼1502 cm^–1^) and the out-of-plane bending vibrations (γC–H, ∼911, 886 cm^–1^) in the fingerprint region, which reflect the substitution pattern, were clearly observed with intensities comparable to those of pure Gen, indicating that the aromatic ring vibrations of Gen were not interfered with by UA. In the spectrum of UA-Gen NPs, the aromatic ring skeleton vibration peak at 1502 cm^–1^ completely disappeared, while the intensities of the fingerprint peaks at 911 and 886 cm^–1^ decreased sharply. The disappearance of aromatic ring skeleton vibration usually indicates a qualitative change in the electron cloud distribution or vibration mode of the aromatic ring. In contrast, the suppression of aromatic ring C–H out-of-plane bending vibration directly reflects that the aromatic ring plane is subject to strong spatial confinement. These changes collectively indicate the occurrence of strong and highly ordered face-to-face π-π stacking between Gen molecules. The molar ratio of two compounds in the nanoparticles was determined to be approximately 5:3 by HPLC. Further molecular dynamics simulations theoretically visualized this process (Figure 2B, C). Guided by the rigid or semirigid framework formed by strong hydrogen bonds and π-π stacking, the triterpenoid skeleton of UA was tightly packed into the voids of the framework through van der Waals forces and hydrophobic interactions, achieving overall dense packing and energy minimization, and thus forming a thermodynamically stable supramolecular assembly with long-range order. X-ray diffraction (XRD) patterns (Figure 2D) showed the disappearance of crystalline diffraction peaks of UA-Gen NPs, indicating that coassembly might lead to the formation of an amorphous state. The amorphous supramolecular structure is maintained by weak intermolecular interactions (hydrogen bonds and hydrophobic interactions), which is deeply coupled with its coassembly mechanism. Furthermore, these interactions are prone to dissociation, which generally facilitates the sustained release of drugs.

**Figure 2:**
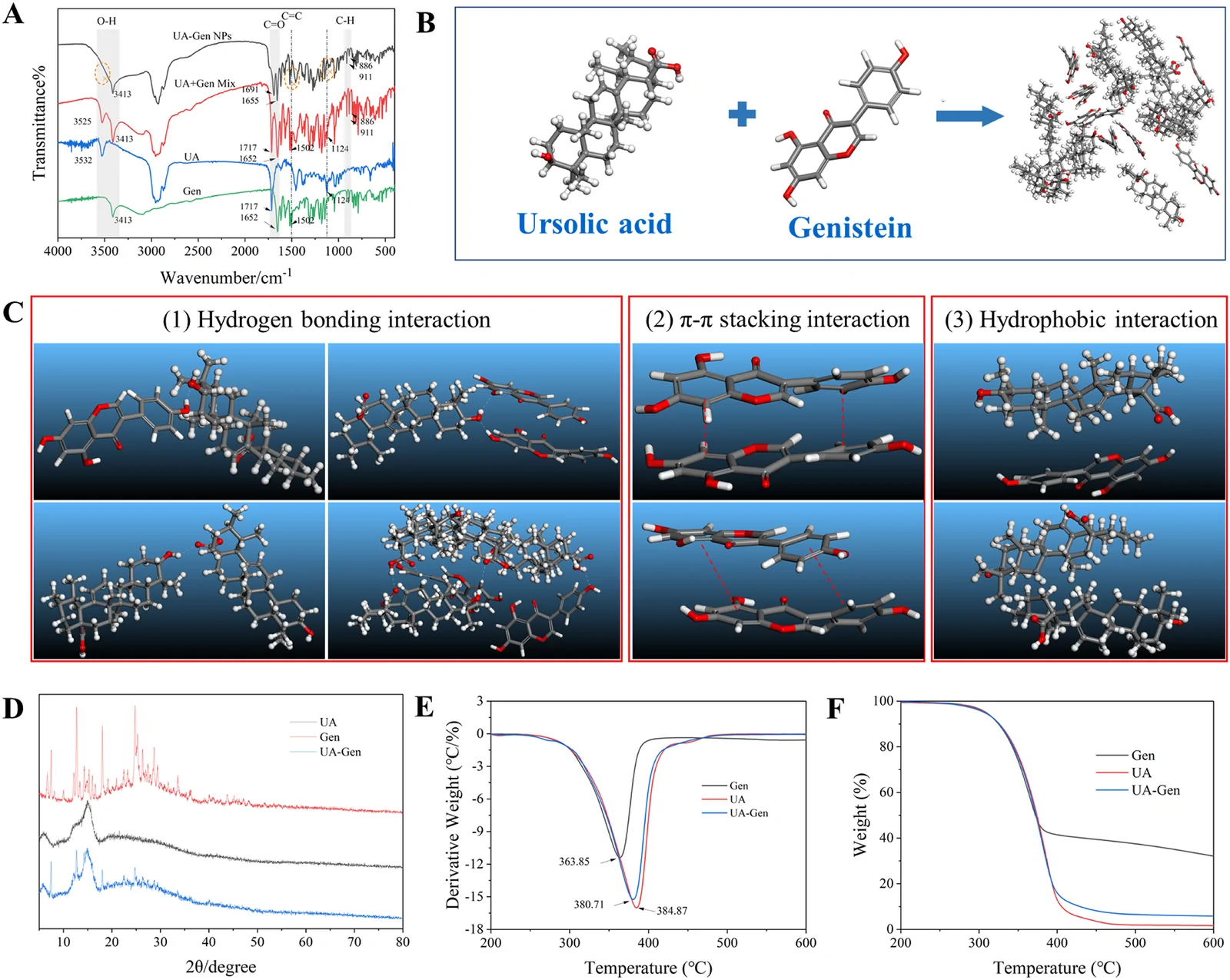
Structural characteristics and intermolecular interaction mechanisms of Gen NPs, UA NPs, and UA-Gen NPs. A. FTIR spectrum of UA-Gen NPs. B. Schematic diagram of molecular assembly: Formation of UA-Gen complex via intermolecular interactions. C. Schematic diagram of molecular interaction simulation: Three types of interactions between UA and Gen: (1) Hydrogen Bond Interaction. (2) π-π Stacking Interaction. (3) Hydrophobic Interaction. D. X-ray diffraction (XRD) patterns of UA, Gen, and UA-Gen complex. E. Derivative thermogravimetric (DTG) curve of UA-Gen NPs (peaks of thermal weight loss rate). F. Thermogravimetric (TG) curve of UA-Gen NPs (trend of thermal weight loss ).

Thermogravimetric analysis/derivative thermogravimetry (TG/DTG) curves (Figure 2E, F) showed that the main weight loss peak of Gen was at 363.85 °C and that of UA at 384.87 °C. In contrast, the main weight loss peak of UA-Gen NPs was at 380.71 °C, falling between those of the two individual components. The thermal decomposition behavior exhibited combined weight loss without the stepwise weight loss pattern of “Gen degradation followed by UA degradation” (i.e., no obvious steps were observed in the TG curve), indicating that UA and Gen were not simply physically mixed but instead formed a novel supramolecular assembly system via intermolecular supramolecular interactions.

However, this study has several limitations. While our strategy aims for self-assembly without external carriers, the iodine-boric acid spectrophotometric method quantified approximately 8.1% of residual PVA in the nanoparticles. Although dichloromethane has high volatility and the sample is freeze-dried, quantitative measurement of dichloromethane residues was not performed in this work.

### 3.3 Responsive Release and In Vivo Pharmacokinetic Analysis of UA-Gen NPs

Therapeutic efficacy is closely associated with the microenvironment-responsive drug release behavior of nanoformulations. We systematically elucidated the intrinsic advantages of UA-Gen NPs as a pH-responsive delivery system from three aspects: drug release characteristics, pharmacokinetic performance, and biocompatibility.

In vitro release studies showed that UA-Gen NPs had distinct pH responsiveness and sequential release characteristics (Figure 3A). At the physiological pH of 7.4, Gen was released rapidly (84.1% at 24 h), while UA exhibited a slow release profile (29.7% at 24 h), indicating the structural stability of the nanoparticles. In the simulated ischemic lesion microenvironment at pH 6.5, the drug release was significantly triggered: Gen achieved nearly complete release within 8 h (98.7%), while the cumulative release rate of UA increased to 53.9% at 24 h, thus forming a sequential release pattern characterized by “rapid Gen release followed by sustained UA release”.

**Figure 3:**
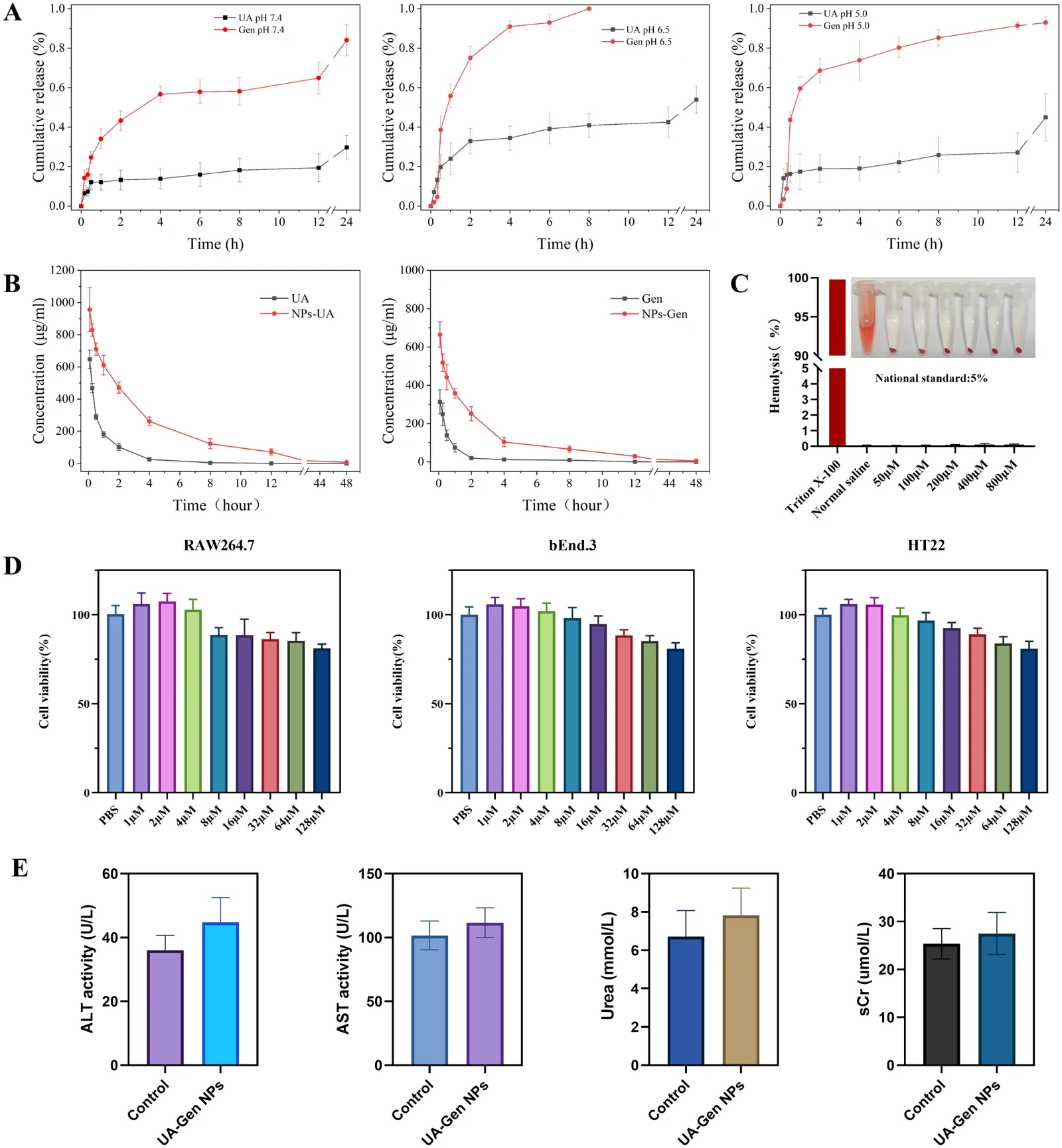
In vitro release profiles and in vivo pharmacokinetic characteristics of UA-Gen NPs. A. In vitro pH-responsive release curves: Release behaviors of UA and Gen under different pH conditions (7.4, 6.5, 5.0). B. Plasma concentration analysis: Left panel shows the concentration–time profiles of UA vs UA-Gen NPs. Right panel shows those of Gen vs Gen NPs. C. Hemolysis rate assay: Hemolysis rates of UA-Gen NPs at different concentrations (50–400 μM) (inset shows experimental samples), with results complying with the biocompatibility standard (<5%). D. Cytotoxicity Assay: Cell viabilities of RAW264.7, bEnd.3 and HT22 cells exposed to UA-Gen NPs (1–128 μM), PBS as control. E. Serum AST, ALT, Urea and sCr levels of mice treated with triple-dose UA-Gen NPs for hepatic and renal safety assessment.

Release kinetic analysis revealed the root of this differentiated behavior from the mechanism. For UA, its release at pH 7.4 conformed to the Fickian diffusion mechanism (the Korsmeyer-Peppas model showed the best fit, n = 0.273), indicating that UA was released via passive diffusion through the pores in the dense structure. When the microenvironment switched to pH 6.5, the optimal model shifted to the first-order kinetic model, which indicated that acidic conditions weakened intermolecular force, resulting in the “relaxation” of the assembly and transforming UA release into a diffusion process driven by its own concentration.

The release of Gen showed higher pH sensitivity and more complex mechanisms. At pH 6.5, its release exhibited an excellent fit with the first-order kinetic model, accompanied by a sharp increase in the release rate constant, which corresponded to its “burst release” behavior. At pH 5.0, its release exponent n was 0.468 (0.43 < *n* < 0.85), which implied the existence of a “structure relaxation-assisted diffusion” mechanism in the supramolecular coassembly system. Specifically, the strongly acidic microenvironment might selectively disrupt the intermolecular interactions surrounding Gen, triggering rapid local structural reorganization and thereby accelerating its diffusion.

Such differential release kinetics indicate that UA and Gen are located in distinct microchemical environments within the coassembled structure. Consequently, the two drugs display temporally and kinetically different release behaviors under identical pH stimulation, forming a staged release pattern. The rapid release of Gen enables immediate intervention against acute inflammation and apoptosis, while the sustained release of UA supports long-term antioxidation and tissue repair.

Pharmacokinetic studies showed (Figure 3B) that UA-Gen NPs improved the in vivo pharmacokinetic characteristics of UA and Gen. Compared with the free UA and Gen, the Areacurve under the concentration–time (AUC_0–∞_) of Gen and UA increased by 6.44-fold and 5.36-fold, respectively. Their peak plasma concentrations (*C*_max_) reached 663.86 ng/L (2.12-fold increase) and 956.03 ng/L (1.48-fold increase), respectively. Meanwhile, the plasma clearance (CL) of the two compounds decreased by 84.5% and 81.4%, which prolonged their mean residence times by 4.67-fold (8.79 h) and 5.50-fold (7.51 h). These results indicate that coassembly improved systemic exposure and partially synchronized the pharmacokinetic profiles of UA and Gen.

A superior therapeutic system must be predicated on safety. Hemolysis assays (Figure 3C) further confirmed that within the concentration range of 50–800 μM, the hemolysis rate remained consistently below 0.2%, which was far superior to the 5% clinical safety threshold, thus verifying its excellent hemocompatibility and fully meeting the requirements for intravenous administration. Cytotoxicity assays (MTT method) showed (Figure 3D) that UA-Gen NPs exerted a concentration-dependent biphasic effect on the viability of RAW264.7 cells, bEnd.3 and HT22 cell lines. The cell viability remained above 80% across all tested concentrations, indicating a broad safety window for therapeutic dosages. Mice were treated with triple therapeutic dose of UA-Gen NPs, and serum AST, ALT, urea and sCr were detected to assess hepatorenal toxicity. No significant changes in renal indicators were found, while only slight elevation of ALT rather than AST was observed, which merely indicated mild hepatocellular stress without severe liver or kidney damage.

These data demonstrate how UA-Gen NPs, as a novel supramolecular coassembled nanotherapeutic platform, systematically address the three core challenges in drug codelivery—controllable drug release, in vivo synchrony, and systemic safety—through their inherent structural and functional advantages, thereby establishing a solid scientific rationale for the improved in vivo therapeutic performance of the nanoformulation relative to the physical mixture of UA and Gen.

### 3.4 UA-Gen NPs Ameliorated Systemic Pathological States and Promote Neurological Function Recovery

Cerebral ischemia-reperfusion injury leads to a series of complex cascading reactions. By comparing the physically mixed drug administration with nanoco-assembly delivery, this study reveals that UA-Gen NPs possess comprehensive and significant advantages in improving neurobehavior, reshaping the redox balance of the organism, and inhibiting excessive inflammatory responses.

Behavioral assessments consistently showed that UA-Gen NPs can most effectively promote the restoration of neurological function. In both the Bederson Behavioral Test (BBT) and the modified Neurological Severity Score (mNSS), the scores of the nanoparticle-treated group were restored to levels close to those of the sham-operated (Control) group, which were significantly superior to those of the physically mixed group (Figure 4A, B). Particularly, in the rotarod test, a method for evaluating complex motor coordination, the nanoparticle group exhibited performance almost comparable to that of the normal group, while the improvement of the mixed group is limited (Figure 4C). These results demonstrate that UA-Gen NPs can not only alleviate the basic neurological injury but also promote the recovery of advanced functions associated with fine motor control, and their comprehensive advantages are attributed to the codelivery of drugs enabled by the nanoparticles.

**Figure 4:**
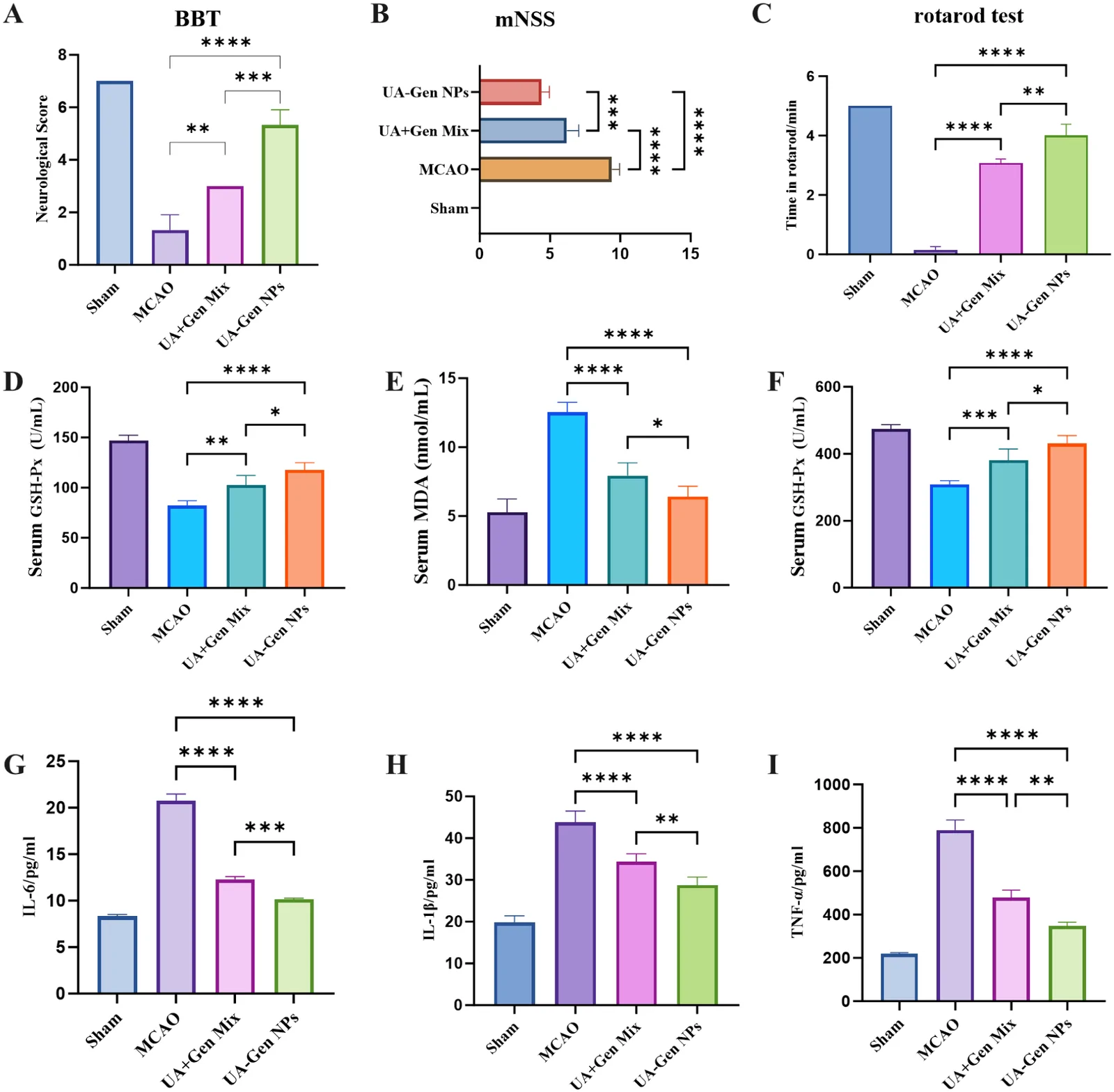
Neuroprotective effects and biochemical regulation of UA-Gen NPs in the MCAO model. A. Bederson Behavioral Test (BBT) score, for evaluating the degree of neurological impairment. B. Modified Neurological Severity Score (mNSS): for quantifying the level of neurological functional impairment in each group. C. Rotarod Test: demonstrating the rotarod retention time of mice in each group, which reflects the differences in motor coordination ability. D–F. Serum Oxidative Stress Markers: showing the levels of SOD (D), MDA (E), and GSH-Px (F) in serum, which reflect the oxidative stress status. G–I. Serum inflammatory cytokine levels: showing the contents of IL-6 (G), IL-1β (H), and TNF-α (I) in serum, which indicate the degree of inflammatory response.

Serological analyses demonstrated that UA-Gen NPs treatment could significantly increase the activities of superoxide dismutase (SOD) and glutathione (GSH), and maximally reduced the level of malondialdehyde (MDA), a lipid peroxidation product, so that all indicators restored to near-normal levels (Figure 4D–F). Although the physical combination group exerted a certain regulatory effect, its efficacy was significantly inferior to that of the nanoparticle group. This proved that the coassembled nanoparticles, via codelivery, significantly enhanced the endogenous antioxidant capacity of the UA-Gen combination, thus more effectively resisting the systemic invasion of oxidative damage.

UA-Gen NPs exhibited potent systemic anti-inflammatory activity, capable of robustly suppressing the levels of key pro-inflammatory cytokines including IL-6, IL-1β and TNF-α to near-normal ranges, with their inhibitory effects significantly superior to those of the physical combination group (Figure 4G–I). This finding profoundly reveals that the therapeutic effect of UA-Gen NPs is not confined to the brain but also regulates the systemic immune response, thereby creating a low-inflammatory systemic environment conducive to neural repair. Its excellent anti-inflammatory effect is mainly attributed to the inhibition of NF-κB-related pathways, while the nanodelivery system further strengthens this pharmacological effect through optimized in vivo drug behavior.

From behavioral assessments to systemic biochemical indices, this section multidimensionally verifies the comprehensive therapeutic advantages of UA-Gen NPs. The recovery of neurological function is founded on the effective suppression of two major pathological processes: oxidative stress and inflammation. The limited improvement observed in the physical combination group across all indicators demonstrates that the inherent combined regulatory potential of the two drugs cannot be fully realized without optimized delivery efficiency. Therefore, UA-Gen NPs successfully transformed the molecular combined pharmacological properties of the two agents into superior in vivo therapeutic benefits via rational coassembly strategy, providing solid phenotypic evidence for further exploring its molecular regulatory mechanism in the brain.

### 3.5 UA-Gen NPs Preserve Blood–Brain Barrier Integrity and Attenuate Parenchymal Ischemic Injury

The progression of cerebral ischemic injury is accompanied by blood–brain barrier disruption, infarct formation, and neuronal death. Subsequently, the structural protective effects of UA-Gen NPs on ischemic brain tissue were evaluated by assessing blood–brain barrier permeability, infarct volume, and histopathological changes.

The Evans blue assay demonstrated (Figure 5A) that the blood–brain barrier was seriously damaged in the model group (MCAO), and the leakage area was about 35%. The Mix treatment partially alleviated the damage (leakage area ≈25%), while UA-Gen NPs showed a significantly stronger protective effect, drastically reducing the leakage area to approximately 10% (Figure 5A). This indicates that nanoparticle codelivery can more effectively preserve barrier integrity, which is closely associated with the combined upregulation of tight junction proteins (e.g., Occludin) and the attenuation of endothelial cell injury.

**Figure 5:**
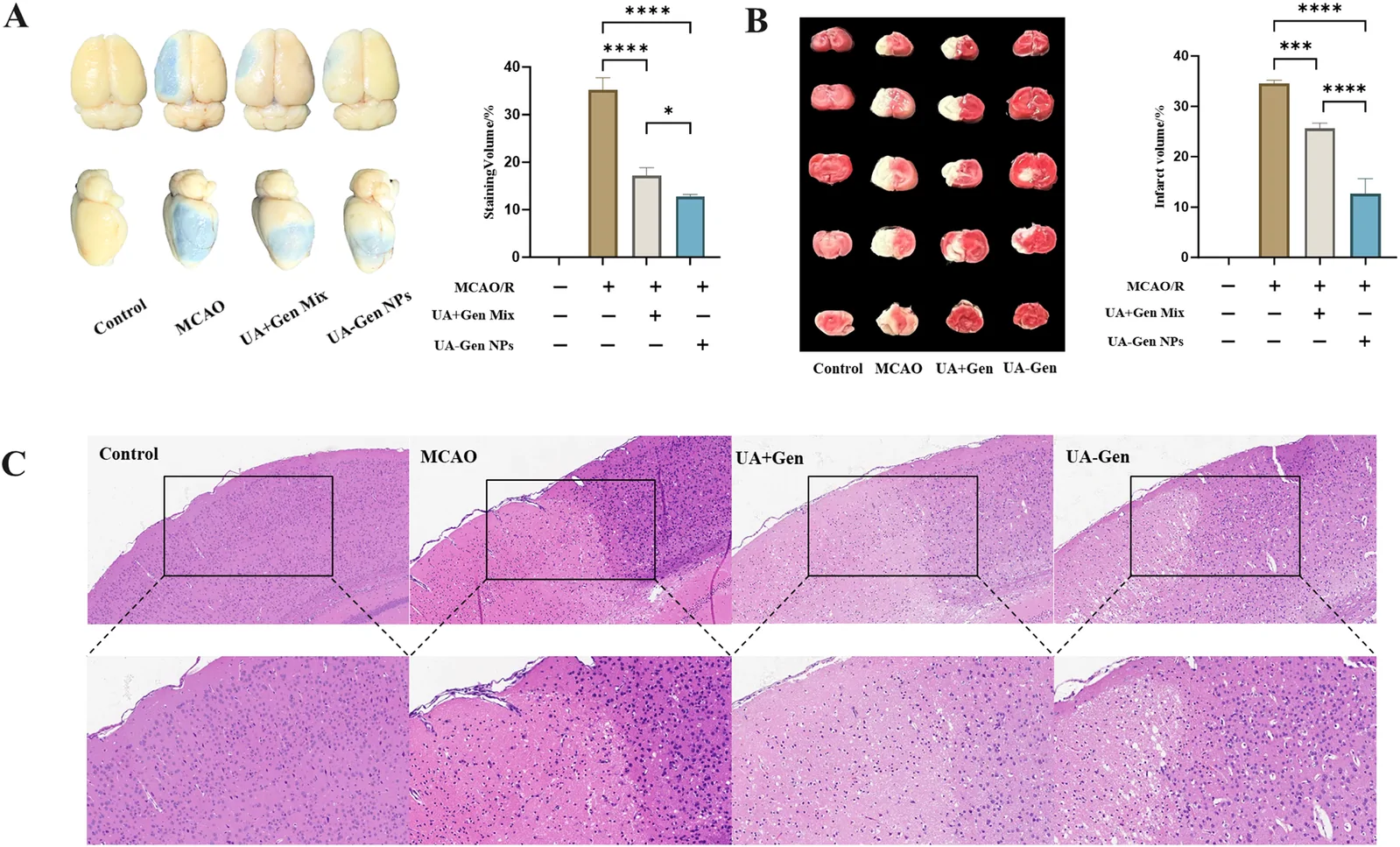
UA-Gen NPs ameliorate blood–brain barrier permeability and alleviate cerebral injury in the MCAO model. A. Evans blue staining was performed to assess blood–brain barrier permeability, and the right panel shows a quantitative bar graph of leakage volume. UA-Gen NPs significantly reduced blood–brain barrier permeability (****P < 0.0001, *P < 0.05). B. 2,3,5-Triphenyltetrazolium chloride (TTC) staining was used to determine cerebral infarct volume, and the quantitative analysis in the right panel demonstrated that UA-Gen NPs significantly reduced the cerebral infarct volume (****P < 0.0001, ***P < 0.001). C. Histopathological observation of brain tissues by H&E staining: H&E-stained sections of brain tissues in each group (upper panels show low-magnification views, while lower panels show high-magnification images of the boxed areas). In the MCAO group, brain tissue cells exhibited disorganization and structural damage, while pathological damage was significantly ameliorated after intervention with UA + Gen and UA-Gen NPs.

The TTC staining results demonstrated (Figure 5B) that the infarct volume in the model group reached approximately 35%. The Mix treatment reduced it to 15–20%, while UA-Gen NPs further diminished the infarct volume to approximately 10%. The gradual reduction in infarct volume was highly consistent with the improved trend of barrier protection, demonstrating that strengthening the blood–brain barrier is a prerequisite for limiting injury expansion and salvaging the ischemic penumbra.

H&E staining provided cytological evidence (Figure 5C). In the model group, neurons exhibited extensive loss, severe morphological damage, and marked edema. The Mix treatment only partially alleviated these pathological changes. In contrast, the brain tissue morphology in the UA-Gen NPs-treated group was further improved: neurons were tightly arranged with plump morphology, and tissue edema was alleviated. These histological observations suggest that the overall cerebral protective effects of UA-Gen NPs may be associated with the maintenance of fundamental neuronal survival and structural integrity.

These findings confirm that UA-Gen NPs exert superior protective effects on the blood–brain barrier, thereby efficiently alleviating cerebral parenchymal infarction and ultimately preserving neuronal morphological integrity. The limited improvements observed in the Mix group across all levels highlight that the absence of a highly efficient delivery system constitutes the bottleneck restricting the exertion of combined therapeutic efficacy. This advantage is inextricably linked to its proven “lockstep” pharmacokinetic profile and sequential drug release characteristics.

### 3.6 Prediction of Multitarget Combinatorial Mechanism Based on Network Pharmacology

Clarifying the combinatorial mechanisms of multicomponent therapeutic systems represents a core challenge in contemporary pharmacological research. Through database screening and comparison, we identified 59 common therapeutic targets of UA, Gen and ischemic stroke (Figure 6A). A protein–protein interaction (PPI) network was further constructed and subjected to topological analysis (Figure 6B), which successfully identified the key regulatory genes located at the core of the network, such as TP53, AKT1, IL6, JUN, and MAPK1. The circular radar chart of gene quantitative indices (Figure 6C) intuitively illustrates the relative importance of these core targets within the network. These hub genes tend to regulate fundamental biological processes including cell apoptosis, inflammatory response, proliferation and survival, suggesting that the combined effects of UA and Gen are likely not mediated by peripheral targets, but rather achieved by intervening in these highly interconnected core nodes that govern cell fate.

**Figure 6:**
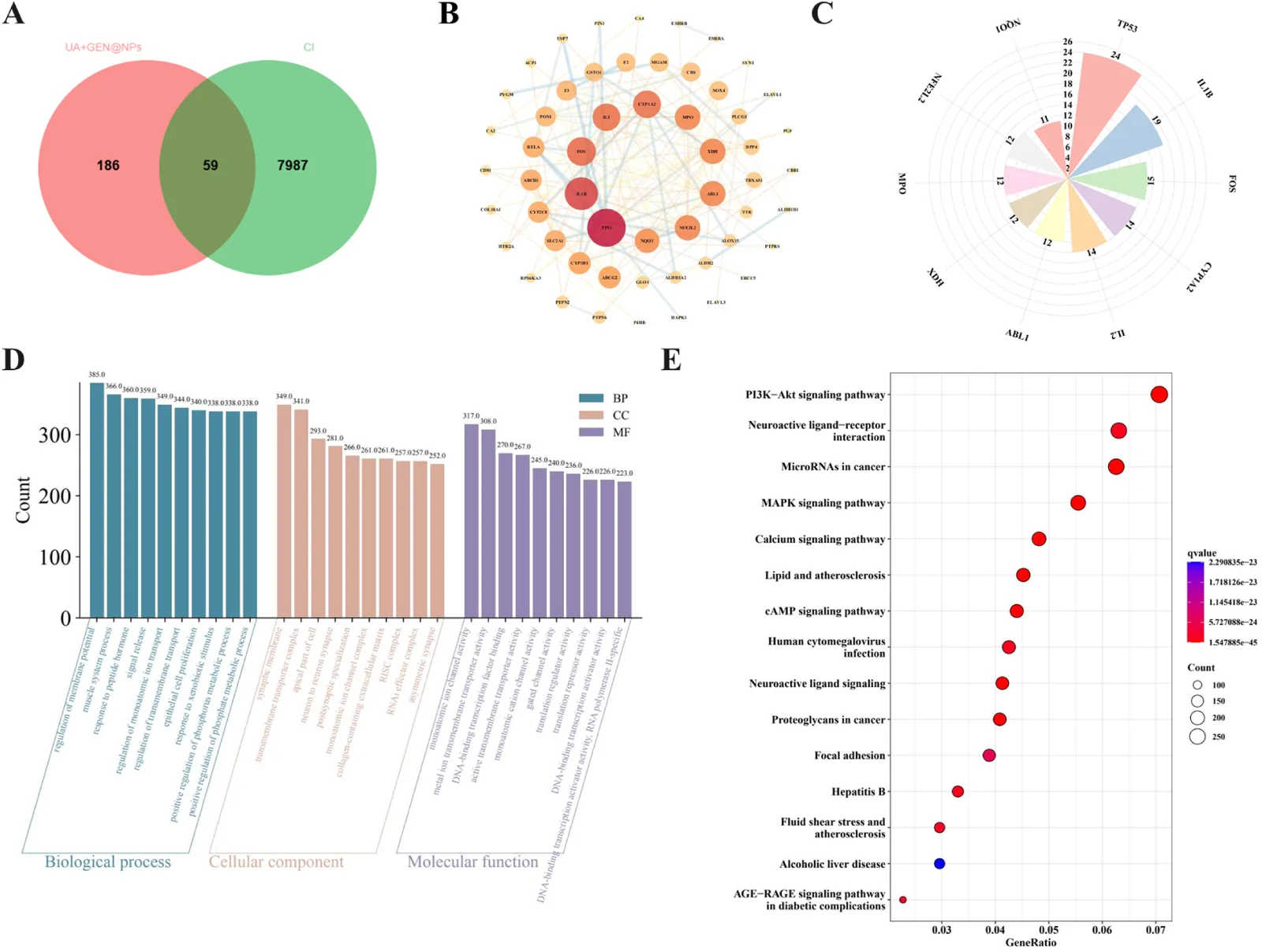
Network pharmacology analysis results of UA and Gen in the treatment of cerebral ischemia. A. Venn diagram of compounds and disease targets. B. Protein–protein interaction (PPI) network analysis diagram: Red nodes represent key regulatory genes. C. Circular radar chart of gene quantitative indices. D. Histogram of GO functional enrichment classification of differentially expressed genes. E. Bubble chart of KEGG pathway enrichment analysis of differentially expressed genes.

GO analysis (Figure 6D) indicated that the intersecting targets were significantly enriched in biological processes including “response to oxidative stress”, “regulation of inflammatory response”, and “negative regulation of apoptotic process”. This highly summarizes the core pathological processes of cerebral ischemia injury from the function and is consistent with the known pharmacological activities of UA and Gen. More directionally, the KEGG pathway enrichment analysis (Figure 6E) provided deeper mechanistic insights. The results demonstrated that the intersecting targets were significantly concentrated in several key signaling pathways, among which the PI3K-Akt, HIF-1, TNF, and Keap1-Nrf2 signaling pathways are particularly prominent. Among these pathways, PI3K-Akt serves as the core pathway regulating cell survival and metabolism.^24,25^ HIF-1 responds to hypoxic stress.^26^ TNF acts as a key initiator of inflammatory storms.^27,28^ while Keap1-Nrf2 functions as the master switch of cellular antioxidant defense.^29,30^ These pathways intersect with each other and collectively form a complex regulatory network in response to ischemic injury. The prediction results indicated that the combined protective effects of UA and Gen might be closely associated with the activation of the PI3K-Akt survival pathway, the inhibition of the TNF inflammatory pathway, and the activation of the Keap1-Nrf2 antioxidant pathway.

### 3.7 Modulation of Keap1-Nrf2 and Occludin Expression by UA-Gen NPs

Based on the prediction results of network pharmacology, we focused on the Keap1-Nrf2 core pathway for experimental validation.

GFAP is a specific marker of astrocytes. Its upregulated expression and morphological changes (from resting to activated states) represent typical stress responses after cerebral ischemia. Excessively activated astrocytes can exacerbate secondary brain injury by forming glial scars and releasing proinflammatory factors.^31,32^ The immunohistochemistry results showed (Figure 7A) that extensive and intense GFAP signals were observed in the model group (MCAO), indicating that ischemic injury induced significant astrocyte proliferation and activation. Physical mixture treatment (UA + Gen) partially alleviated such excessive astrocyte activation. In contrast, the intensity and range of GFAP-positive signals in the UA-Gen NPs treatment group were maximally suppressed. This indicated that nanoparticle codelivery could redirect astrocyte responses toward a more reparative phenotype, thus providing a crucial cytological basis for its observed superior neuroprotective effects.

**Figure 7:**
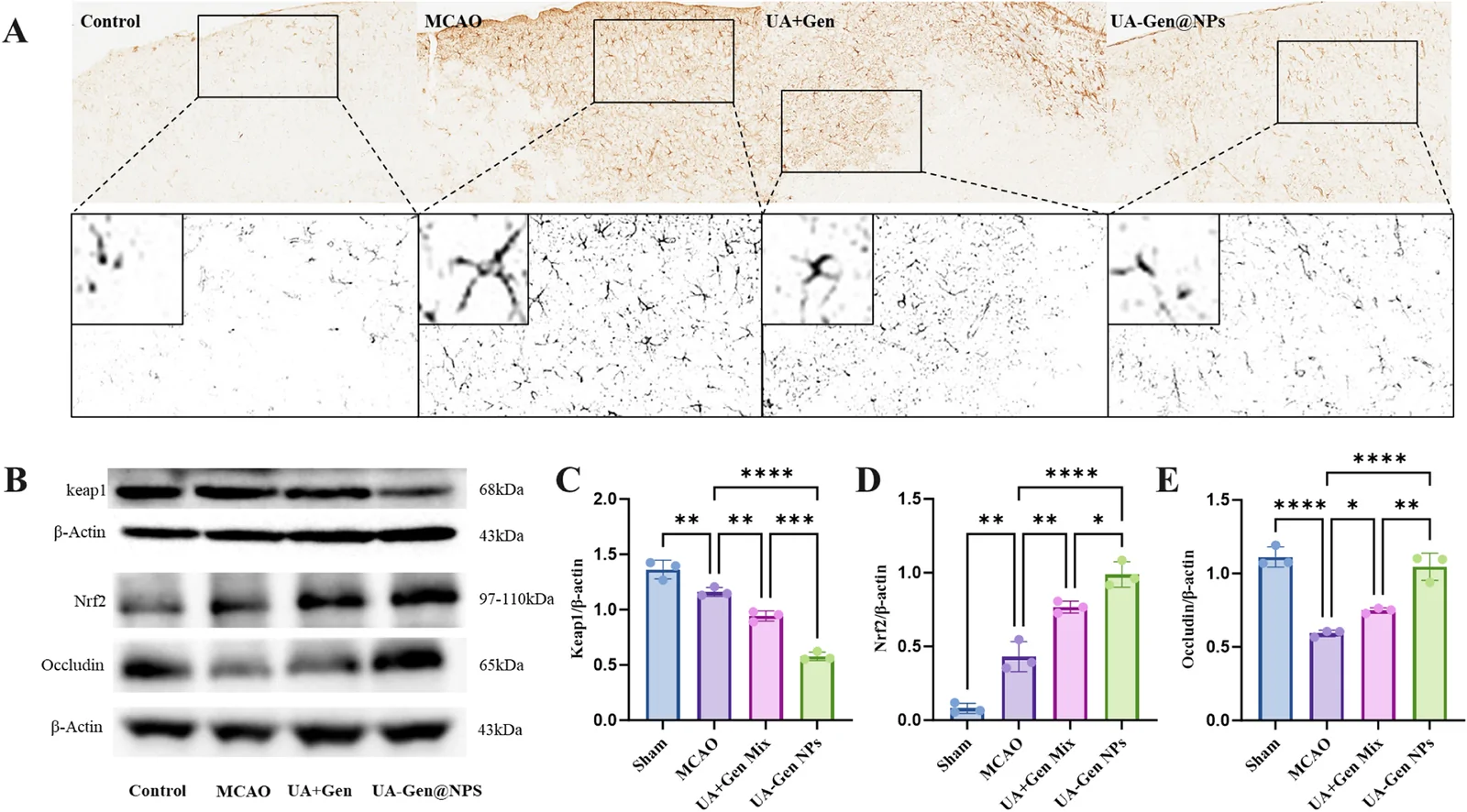
Astrocyte activation and expression profiles of Nrf2 pathway- and blood–brain barrier-related proteins in the brain injury model. A. Immunohistochemical staining of glial fibrillary acidic protein (GFAP, a specific marker of astrocytes) in brain tissues. B. Protein expression bands of Keap1, Nrf2, Occludin, and internal reference β-Actin in brain tissues of each group. C–E. Quantitative bar graphs of protein expression: statistical analysis of relative expression levels of Keap1/β-Actin (C), Nrf2/β-Actin (D), and Occludin/β-Actin (E), respectively (*P < 0.05, **P < 0.01, ***P < 0.001, ****P < 0.0001).

In order to explain the molecular driving force of the above-mentioned cellular phenotypic changes, we detected the key proteins of the core predicted pathway, namely the Keap1-Nrf2 axis. Under physiological conditions, Keap1 maintained the low expression of Nrf2 by promoting its degradation. In response to cerebral ischemic stress, the downregulation of Keap1 expression can relieve the inhibition of Nrf2, thereby activating the transcription of antioxidant genes.^33,34^

Western Blot analysis provided evidence for the core molecular mechanism (Figure 7B–E). Compared with the sham operation group, the expression of Keap1—the inhibitory protein of Nrf2—was significantly decreased in the model group, whereas the expression of Nrf2 itself was compensatorily upregulated. This reflected an attempt to initiate the endogenous antioxidant program after ischemic stress, which, however, was insufficient to counteract the severe injury. UA-Gen NPs treatment induced the most robust beneficial alterations in this pathway, as it most significantly downregulated Keap1 expression and, concomitantly, most potently upregulated the total protein level of Nrf2 (Figure 7C, D). This amplification effect of “Keap1 inhibition-Nrf2 activation” was significantly stronger than that observed in the physical mixture group. As the master transcription factor of cellular antioxidant responses, the robust activation of Nrf2 implies a comprehensive enhancement in the transcription of a series of downstream phase II detoxifying enzymes and antioxidant proteins. This directly explains, from a molecular mechanism perspective, why the UA-Gen NPs treatment group exhibited the strongest systemic antioxidant capacity in the animal model (Figure 4D–F).

Notably, the expression of Occludin, a core tight junction protein, was severely impaired in the model group, whereas its expression was restored following treatment (Figure 7B, E). Crucially, UA-Gen NPs has the most significant up-regulation effect on Occludin, and its recovery level exceeds that of the physical mixture group and it is positively correlated with the activation level of Nrf2. This provides conclusive molecular evidence for the effective blood–brain barrier protective effect of nanoparticles observed in Figure 5A. The consistent alterations in the protein expression levels of Keap1, Nrf2, and Occludin constitute a clear “signal input-core regulation-functional output” axis.

This part of the study not only confirmed the network pharmacology prediction, but also revealed the superior neuroprotective effect of UA-Gen NPs, which was achieved by amplifying a core hub pathway (Keap1-Nrf2), and then improving oxidative stress, neuroinflammation and the integrity of blood–brain barrier in multiple directions. This mechanism of “one-node activation yielding multidimensional benefits” highlights the substantial advantages and therapeutic potential of combined-agent drug design targeting core regulatory nodes.

## 4 Conclusion

In this study, via rational supramolecular design, UA and Gen were directly assembled into pH-responsive nanotherapeutic entities (UA-Gen NPs) with uniform morphologies. This strategy establishes an elegant reconstruction paradigm where the active drug molecules inherently serve as the building blocks. By striving to minimize dependence on exogenous carrier-building materials, this strategy can largely circumvent the structural complexity and potential toxicity associated with conventional delivery carriers. Functionally, UA-Gen NPs not only ensure the synchronized systemic circulation of both drugs but also enable a response to the acidic microenvironment of ischemic lesions, triggering on-demand, programmed sequential drug release. This upgrades the pharmacological intervention from static tissue coverage to dynamic pathological management. Therapeutically, this nanosystem efficiently activates Keap1-Nrf2, a core hub pathway for cellular defense, thereby amplifying the body’s intrinsic network responses including antioxidation, anti-inflammation, and barrier repair. This indicates a shift in therapeutic strategy, from an exogenous confrontation to a systemic enhancement of endogenous protective ability.

In summary, this research successfully reshapes a simple physical drug mixture into a multifunctional, highly integrated nanosystem. The main advantages of this platform lie in the effective integration of molecular self-assembly, sequential pH-responsive drug release, and multitarget network modulation. By highlighting rational design based on molecular interactions and responses to the pathological microenvironment, this study paves a highly promising path for next-generation therapies against ischemic stroke and other complex diseases.

## References

[1] Wang H., Lin J., Zhang S., Zhao F., Zhang X., Wang L., Zhang C., Yin Y., Luo R., Hu R. (2025). Global, Regional,
and National Burden of Stroke and Its Subtypes: Unravelling the Correlations
with the Global Aging Trend. Neuroepidemiology.

[2] Chen X., Lu L., Xiao C., Lan Y., Zhong S., Qin C., Tang Y. (2025). Global burden of ischemic stroke in adults aged 60 years and older
from 1990 to 2021: Population-based study. PLoS
One.

[3] Lapchak P. A. (2012). A series
of novel neuroprotective blood brain barrier penetrating flavonoid
drugs to treat acute ischemic stroke. Curr.
Pharm. Des..

[4] Zhang S., Zhou Y., Li R., Chen Z., Fan X. (2022). Advanced drug
delivery system against ischemic stroke. J.
Controlled Release.

[5] Jin X., Liu S., Chen S., Han R., Sun X., Wei M., Chang Y., Li L., Zhang H. (2025). Small-molecule probes
based on natural products: Elucidation of drug-target mechanisms in
stroke. J. Pharm. Anal..

[6] Gugliandolo A., Bramanti P., Mazzon E. (2020). Activation
of Nrf2 by Natural Bioactive
Compounds: A Promising Approach for Stroke?. Int. J. Mol. Sci..

[7] Liu J., Zhang X., Guo J., Zhang Y., Fan J., Liu J., Chen J., Jiang J., Yu B., Zhang K. (2025). Ursolic acid ameliorates cerebral ischemia-reperfusion injury by
inhibiting NF-κB/NLRP3-mediated microglia pyroptosis and neuroinflammation. Front. Pharmacol..

[8] Schreihofer D. A., Oppong-Gyebi A. (2019). Genistein:
mechanisms of action for a pleiotropic neuroprotective
agent in stroke. Nutr. Neurosci..

[9] Liang H. W., Qiu S. F., Shen J., Sun L. N., Wang J. Y., Bruce I. C., Xia Q. (2008). Genistein
attenuates oxidative stress
and neuronal damage following transient global cerebral ischemia in
rat hippocampus. Neurosci. Lett..

[10] Feng W., Yang K., Zou Y., Xiao Z., Qian R., Qian R. (2025). Progress of ursolic acid on the regulation of macrophage: summary
and prospect. Front. Immunol..

[11] Mlala S., Oyedeji A. O., Gondwe M., Oyedeji O. O. (2019). Ursolic
Acid and
Its Derivatives as Bioactive Agents. Molecules.

[12] Motawea A., Maria S. N., Maria D. N., Jablonski M. M., Ibrahim M. M. (2024). Genistein transfersome-embedded topical
delivery system
for skin melanoma treatment: in vitro and ex vivo evaluations. Drug Delivery.

[13] Li Y. X., Wang H. B., Jin J. B., Yang C. L., Hu J. B., Li J. (2022). Advances in
the research of nano delivery systems in ischemic stroke. Front. Bioeng. Biotechnol..

[14] Li C., Yao S., Li Z., Gao Y. (2025). Application of Novel
Drug-Delivery
Strategies in Neurological Disorders. Adv. Mater..

[15] Zhang Y. B., Lian Y. J., Zhou C. L., Cheng J. J., Zhao S., Liu H. J., Wang J. C., Lu X., Shi J. H., Du G. H. (2025). Self-assembled natural triterpenoids
for the delivery of cyclin-dependent
kinase 4/6 inhibitors to enhance cancer chemoimmunotherapy. J. Controlled Release.

[16] Fu S., Li G., Zang W., Zhou X., Shi K., Zhai Y. (2022). Pure drug
nano-assemblies: A facile carrier-free nanoplatform for efficient
cancer therapy. Acta Pharm. Sin. B.

[17] Zhou M., Han S., Aras O., An F. (2021). Recent Advances in Paclitaxel-based
Self-Delivery Nanomedicine for Cancer Therapy. Curr. Med. Chem..

[18] Zhou C. L., Zhao S., Zhang Y. B., Cheng J. J., Shi J. H., Du G. H. (2024). Mesoporous polydopamine
Targeting CDK4/6 Inhibitor toward Brilliant
Synergistic Immunotherapy of Breast Cancer. Small.

[19] Zhang X., Li N., Zhang S., Sun B., Chen Q., He Z., Luo C., Sun J. (2020). Emerging carrier-free
nanosystems based on molecular
self-assembly of pure drugs for cancer therapy. Med. Res. Rev..

[20] Wang J., Qiao W., Li X., Zhao H., Zhang H., Dong A., Yang X. (2021). A directed
co-assembly of herbal
small molecules into carrier-free nanodrugs for enhanced synergistic
antitumor efficacy. J. Mater. Chem. B.

[21] Qu S., Wang T., Liu J., Qin J., Yang B., Liu Y., Li P., Wang G., Ling F. (2026). A Computational Strategy
for Identifying Self-Assembling Food-Derived Molecules for Antiparasitic
Nanotherapy. Adv. Sci..

[22] Luo X., Zhao C., Yu F., Dong X., Hao Y., Zhang H., Liu B., Jiang Y. (2026). Targeted restoration
of macrophage mitochondrial function by astaxanthin-geniposide co-assembled
nanosystem for atherosclerosis treatment. Chem.
Eng. J..

[23] Peng H., Li H., Lin J., Li L., Li M., Huang H., Wu J., Chen B. (2026). Carrier-free
targeted co-assembly of glycyrrhizin and
puerarin overcomes ocular barriers for corneal alkali burn therapy
via macrophage reprogramming. Mater. Today Bio.

[24] Liu T., Wang W., Li X., Chen Y., Mu F., Wen A., Liu M., Ding Y. (2023). Advances of phytotherapy in ischemic
stroke targeting PI3K/Akt signaling. Phytother.
Res..

[25] Guo H., Li Y., Wang S., Yang Y., Xu T., Zhao J., Wang J., Zuo W., Wang P., Zhao G., Wang H., Hou W., Dong H., Cai Y. (2024). Dysfunction
of astrocytic glycophagy exacerbates reperfusion injury in ischemic
stroke. Redox Biol..

[26] Tsao C. C., Baumann J., Huang S. F., Kindler D., Schroeter A., Kachappilly N., Gassmann M., Rudin M., Ogunshola O. O. (2021). Pericyte
hypoxia-inducible factor-1 (HIF-1) drives blood-brain barrier disruption
and impacts acute ischemic stroke outcome. Angiogenesis.

[27] Fanzhou R., Yuding L., Pingchuan L., Hai X., Xu L., Jian W. (2025). The role of TNF signaling pathway in post-stroke cognitive impairment:
a systematic review. Ann. Med..

[28] Gu L., Wu G., Long J., Su L., Yan Y., Chen Q., Xie J., Hu Y. (2013). The role of
TNF-α 308G > A polymorphism in the
risk for ischemic stroke. Am. J. Med. Sci..

[29] Pant A., Dasgupta D., Tripathi A., Pyaram K. (2023). Beyond Antioxidation:
Keap1-Nrf2 in the Development and Effector Functions of Adaptive Immune
Cells. Immunohorizons.

[30] Yamamoto M., Kensler T. W., Motohashi H. (2018). The KEAP1-NRF2
System: a Thiol-Based
Sensor-Effector Apparatus for Maintaining Redox Homeostasis. Physiol. Rev..

[31] Clifford T., Finkel Z., Rodriguez B., Joseph A., Cai L. (2023). Current Advancements
in Spinal Cord Injury Research-Glial Scar Formation and Neural Regeneration. Cells.

[32] Gollihue J. L., Norris C. M. (2020). Astrocyte mitochondria: Central players and potential
therapeutic targets for neurodegenerative diseases and injury. Ageing Res. Rev..

[33] Wang L., Zhang X., Xiong X., Zhu H., Chen R., Zhang S., Chen G., Jian Z. (2022). Nrf2 Regulates Oxidative
Stress and Its Role in Cerebral Ischemic Stroke. Antioxidants.

[34] Alfieri A., Srivastava S., Siow R. C., Modo M., Fraser P. A., Mann G. E. (2011). Targeting the Nrf2-Keap1 antioxidant defence pathway
for neurovascular protection in stroke. J. Physiol..

